# Phenotypic Variability in a Coinfection With Three Independent *Candida parapsilosis* Lineages

**DOI:** 10.3389/fmicb.2020.01994

**Published:** 2020-08-27

**Authors:** Emilia Gómez-Molero, Jesse R. Willis, Anna Dudakova, Laia Carreté, Michael Weig, Uwe Groß, Attila Gácser, Toni Gabaldón, Oliver Bader

**Affiliations:** ^1^Institute for Medical Microbiology, University Medical Center Göttingen, Göttingen, Germany; ^2^Comparative Genomics Group, CRG-Centre for Genomic Regulation, Barcelona, Spain; ^3^Department of Microbiology, University of Szeged Interdisciplinary Excellence Centre, Szeged, Hungary; ^4^MTA-SZTE Lendület Mycobiome Research Group, University of Szeged, Szeged, Hungary; ^5^Universitat Pompeu Fabra (UPF), Barcelona, Spain; ^6^ICREA, Barcelona, Spain

**Keywords:** *Candida parapsilosis*, coinfection, genome sequencing, phenotypic variation, adhesin genes

## Abstract

The human pathogenic yeast *Candida parapsilosis* has gained significant importance over the past decades as one of the principal causes of fungal bloodstream infections. Isolates of *C. parapsilosis* are known to be able to switch between several different colony morphologies in vitro, which are correlated with different cell shapes, altered cell surface properties, and thus different capacities to form biofilms on indwelling medical devices. In a set of six clinical specimens from a single surgery patient yielding stable smooth- as well as crepe-morphology isolates, we investigated the differences between five of them on a phenotypic and genomic level. In contrast to the initial assumption that they were switched forms of a clonal strain, karyotyping and genome sequencing showed that the patient was colonized by at least three distinct linages. Statistical analysis placed these groups distantly across the population of *C. parapsilosis*. Interestingly the single blood culture isolate was of smooth morphology and matched with an isolate from the patient’s nose of similar morphology. Strong variation between the isolates was seen in adhesin-encoding genes, where repeat regions showed significant variation in length and repeat-numbers, most strikingly in *HWP1* of the smooth isolates. Although no differences in drug susceptibility were evident, the high phylogenetic distance separating the individual strains highlights the need for testing of multiple colonies in routine practice. The absence of biofilm formation in the blood stream isolate indicates a lack of respective adhesins in the cell wall, in turn pointing toward lack of adhesion as a positively contributing factor for dissemination.

## Introduction

Phenotypic variation is a common phenomenon among *Candida* species. In *Candida parapsilosis* at least five different colony morphologies (“smooth”, “snowball”, “rough”, “crepe”, and “concentric”) have been described and partially been associated with different cellular shapes (Lott et al., 1993; Enger et al., 2001; Laffey and Butler, 2005; Nosek et al., 2009) ranging from yeast form to pseudohyphal growth, and having different cell sizes. In particular, the different cellular shapes have been found to display altered aggregation and adhesion phenotypes, a fact that may explain the different colony morphologies. Its reference genome CDC-317 encodes at least five different adhesion proteins which can vary both in presence and in structure between different isolates (Butler et al., 2009; Pryszcz et al., 2013).

*In vitro* transition between colony morphologies has been observed at frequencies at around 10^–2^ to 10^–4^ (Laffey and Butler, 2005) and can be visualized through different chemical stimuli such as the presence of different amino acids (Kim et al., 2006) or Phloxine B (Laffey and Butler, 2005). Although the biofilm formation capacities of *C. parapsilosis* isolates have been shown to correlate with increased mortality in patients with *C. parapsilosis* sepsis (Tumbarello et al., 2012; Soldini et al., 2018), it is unclear how the different morphologies reflect this in the clinical laboratory.

**FIGURE 1:**
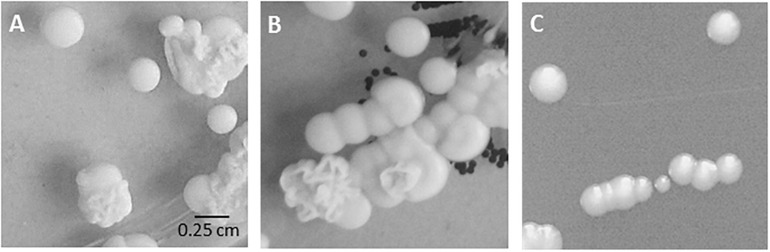
Colony morphology in routine cultures. Mixed fungal growth on original Sabouraud’s agar plates from **(A)** nose swab (specimen 1701), **(B)** throat swab (specimen 1702). **(C)** Isolate bsc-1700 (blood culture, smooth colony, specimen 1700) on Sabouraud’s agar plate.

During our diagnostic procedures, we were faced with specimens yielding a series of *C. parapsilosis* sensu stricto isolates, culminating in isolation of *C. parapsilosis* from blood and from the patient’s CVC (Table 1). The screening specimen obtained from nose and throat each showed a mixture of smooth and wrinkly colony morphologies of *C. parapsilosis* while the blood culture specimen produced only smooth-type colonies (Figure 1).

**TABLE 1:** Time course of interventions and laboratory findings.

Day	Intervention	Laboratory result
1	Lung surgery	
3	BAL taken	Yeast colonies
11	Caspofungin initiated	
12	Bronchial secretion taken	Yeast colonies
	Blood culture pair taken	Culture negative
	CVC exchanged	Culture negative
16	Blood culture pair 1700 taken^a^	*C. parapsilosis*
19	reported “*C. parapsilosis”* to physician for blood culture	
20	Reported azole susceptible for blood culture isolate	
	Nose swab 1701 taken	*C. parapsilosis*
	Throat swab 1702 taken	*C. parapsilosis*
21	i.v. fluconazole therapy initiated	
26	CVC exchanged	Culture negative
	Caspofungin therapy stopped	
30	CVC exchanged	*C. parapsilosis*
34	CVC exchanged	Culture negative
41	Fluconazole therapy stopped after disappearance of clinical symptoms	
43	CVC removed	Culture negative
45	Patient discharged	

*BAL, bronchoalveolar lavage; CVC, central venous catheter. ^*a*^all subsequent blood cultures taken at 2–3 days intervals remained negative for fungi.*

*C. parapsilosis* is known to be able to switch between different cellular shapes and, as a consequence of concomitantly altered cell surface properties, between different colony morphologies. Such switching events have been observed *in vitro* at varying frequencies (Lott et al., 1993; Enger et al., 2001; Laffey and Butler, 2005). Stages with non-smooth morphologies have been described to have increased biofilm formation capacity and virulence (Tumbarello et al., 2012; Soldini et al., 2018).

The aim of the study described here was to quantify the phenotypic variation and the degree of relatedness between the five morphologically different isolates from the same patient, and to reflect on the potential consequences for the treatment course.

## Results

### Patient History Workup

The elderly patient had been admitted to the intensive care unit for observation with post-surgical complications, including sepsis, after diagnostic lung surgery and received antibacterial treatment since 3 days before ICU admission and had been given antifungal therapy (day 11) for suspected fungal infection as the fever did not remit under antibacterial treatment. Routine diagnostic drug susceptibility testing showed all fungal isolates to be susceptible to routinely used antifungal drugs over the entire course of treatment. The patient was treated first with caspofungin, and once *C. parapsilosis* was diagnosed also with fluconazole; later antifungal therapy was switched to fluconazole-only with favorable outcome: after 48 d ICU stay, organ functions were stabilized, the replacement CVC remained without cultural findings after removal, and the patient was eventually discharged (Table 1).

### Routine Diagnostics

The fungal growth from BC, swabs, and CVC was identified by MALDI-TOF as *Candida parapsilosis* sensu stricto (Table 2), independently of the colony morphology. Yeasts from BAL, bronchial secrete, and CVC were unfortunately discarded prior to the study, and not available for further analyses. Isolates included in the study were given acronyms indicating the source (*b* = blood, *n* = nose, *t* = throat) as well as the colony phenotype (sc = smooth colony, cc = crepe colony). The blood culture isolate bsc-1700 was one log_2_-fold decreased in susceptibility toward micafungin and caspofungin as compared to other isolates (Table 2), but not considered resistant.

**TABLE 2:** Isolate characteristics.

	Study isolates						Reference isolates				
Strain acronym	bsc-1700	nsc-1701	ncc-1701	tsc-1702	tcc-1702	cvc	CDC-317	ATCC 22019	CBS 1954	CBS 6318	GA-1
Deposition number	DSM 108633	DSM 108634	DSM 108636	DSM 108635	DSM 108637	n. a.^a^	ATCC MYA-4646	CBS 604, DSM 5784	ATCC 28474	ATCC 7330	SZMC 8110
SRA	SAMN10782958	SAMN10782959	SAMN10782961	SAMN10782960	SAMN10782962	n. a.	n. a.	n. a.	n. a.	n. a.	n. a.
Specimen	Blood culture	Nose swab	Nose swab	Throat swab	Throat swab	CVC	Skin	Sprue	Environ mental	Skin	Blood culture
Colony morphology	Smooth	Smooth	Crepe	Smooth	Crepe	n. d.	Smooth	Concentric	Crepe	Smooth	Smooth
MBT^c^	2.020	2.020	2.050	2.070	2.103	2.201	n. d.	n. d.	n. d.	n. d.	n. d.
**Drug susceptibility^b^**											
FLZ (mg/L)	0.5	0.5	0.5	0.5	0.25-1	S	4	1	1	1	0.5
PSZ (mg/L)	0.032	0.032–0.064	0.016–0.032	0.032	0.032	n. d.	0.032	0.032	0.032	0.032	0.032
VRZ (mg/L)	0.032	0.032	0.032	0.032	0.032	S	0.125	0.032	0.032	0.032	0.032
CAF (mg/L)	0.5	0.25–0.5	0.5	0.25–0.5	0.25–1	S	1	0.5	0.5	0.5	1
MIF (mg/L)	2	1–2	1–2	1–2	1–2	S	2	1	2	2	2
5FC (mg/L)	0.032	0.032–0.064	0.064–0.125	0.032–0.064	0.064–0.125	n. d.	0.032	0.250	0.125	0.032	0.125

*^*a*^Isolate not preserved, values from routine diagnostics. ^*b*^all EUCAST broth microdilution, except isolate cvc (there: VITEK II). C MBT: log score value issued by the MALDI Biotyper system (Bruker Daltonics); values above 1.999 reflect species-level identifications. n. a., not applicable; n. d., not determined.*

### Isolates Vary in Adhesin-Related Phenotypes

**FIGURE 2:**
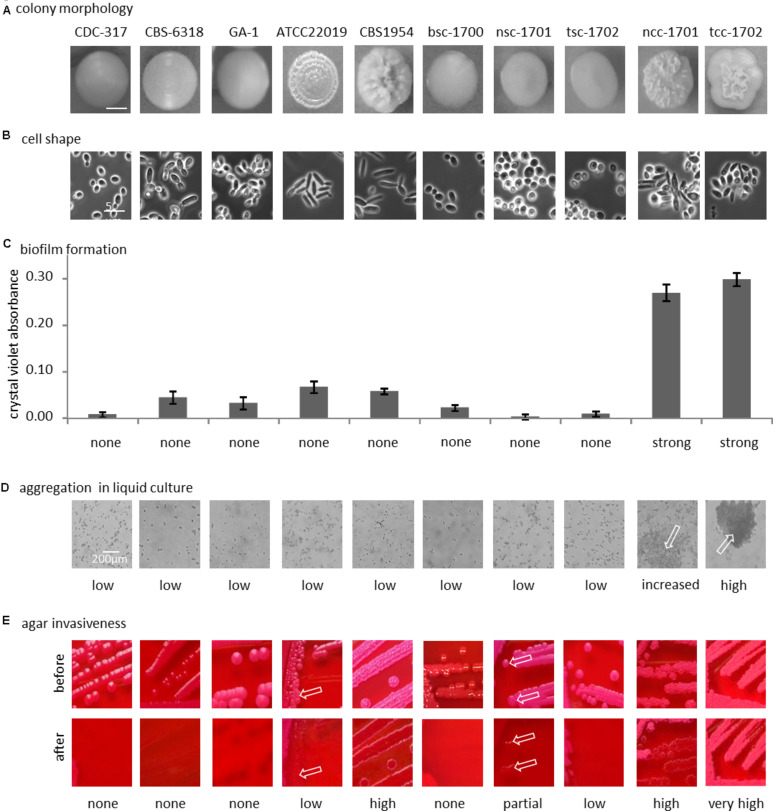
Phenotypical differences. **(A)** Colony morphology on phloxine B agar; bottom row, cellular shape (100-fold magnification, phase contrast). **(B)** Representative morphology of the colony used below in panels **(C–E)**. **(C)** Biofilm formation on polystyrene. **(D)** Clumping of cells in liquid culture (optical microscopy, 40x magnification), arrows indicate clumps. **(E)** Agar invasion, arrows indicate small localized sites of agar invasion in otherwise non-invasive strain. See text for details.

After passaging on Phloxine B agar, morphologically stable “smooth” and “crepe” morphotypes were obtained from both swab specimens. The blood culture yielded only a “smooth” morphotype (Figure 2A).

With the exception of control strain CBS 6318, cells from smooth colonies microscopically appeared as round yeast cells (Figure 2B). Cultures of CBS 6318 additionally contained a large proportion of stretched yeast-like cells. Cells from colonies with increasing wrinkly appearance were more likely to appear as pseudohyphae, with CBS 1954 having the strongest colony phenotype and pure pseudohyphal cells. Crepe colonies isolated from our patient contained a mixture of both, yeast-form and pseudohyphal cells, this phenotypic mixture being stable over at least 10 additional passages on solid media.

Biofilm formation on polystyrene was not strictly correlated with morphotype, but exceedingly high in the two crepe strains ncc-1701 and tcc-1702. In comparison, it was only basal in the reference isolates, independently of their morphotypes (Figure 2C). In addition, the blood culture isolate did not display increased biofilm formation (Figure 2C).

Only the two crepe strains aggregated when cultured in liquid medium (Figure 2D). In contrast, at least partial agar invasion was evident with all non-smooth strains (Figure 2E). Otherwise, agar invasion was observed most strongly in strains with pseudohyphal growth. In the case of the “clumpiest” isolate tcc-1702 the colonies were not removed from the agar by running water (Figure 2E). As compared to tcc-1701, this suggests a stronger cohesion of the colonies in addition to the already increased agar invasiveness.

In summary, the smooth isolates bsc-1700, nsc-1701, and tsc-1702 were phenotypically highly similar, while among the two crepe isolates, tcc-1702 displayed stronger cell-cell aggregation than ncc-1701.

### The Five Isolates Fall Into Three Genetically Distinct Groups

**FIGURE 3:**
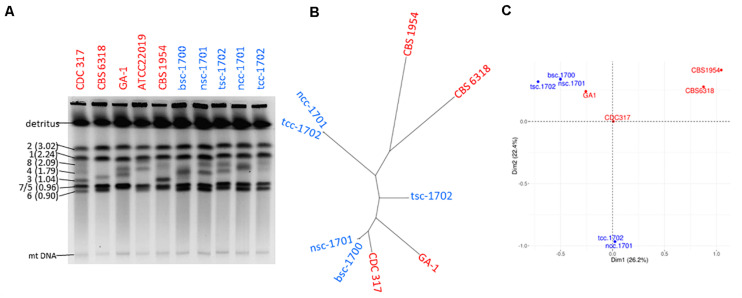
Strain typing and genome sequencing. **(A)** Pulse-field gel electrophoresis suggests the five isolates (blue) are not clonal. **(B)** Distance-based and **(C)** MCA cluster analysis of the SNPome placing the 5 patient isolates into the context of other genome-sequenced strains (red), relative to CDC-317. Both indicate the simultaneous presence of at least three independent clonal groups in the patient.

Genetic relatedness of the isolates was therefore addressed first by karyotyping to visualize potential chromosomal changes. This revealed clear variations among medium sized chromosomes (presumptively Chr8, 4, and 3) between all patient as well as control isolates. The chromosomal pattern of the blood culture isolate bsc-1700 appeared most similar to the nose crepe isolate ncc-1701 (Figure 3A), but not identical.

Whole genome sequencing detected a total of 3846 single nucleotide polymorphisms (SNPs) among the isolates as compared to the reference CDC-317 (Figure 3B) whose distribution suggested them to belong to three disparate groups (Figure 3B): the two crepe isolates ncc-1701 and tcc-1702 were near clonality (>99% shared SNPs), as were bsc-1700 and nsc-1701 (>98% shared SNPs). Still, each member of a clonal pair contained private SNPs, so that each pair was likely progeny of a different common ancestor. Both clonal pairs were most distant from each other with <15% of shared SNPs. Strain tsc-1702 was a genetic outsider (45-68% shared SNPs with the other groups). A PCA-based population analysis including other available genome sequences suggested a genetic distance between the three clusters as far as with independent controls (Figure 3C).

### Antifungal Drug Susceptibility

Since the MIC values toward echinocandins (Table 2) were bordering the resistance level, we analyzed the potential echinocandin resistance genes CPAR2_804030, CPAR2_109680, and CPAR2_106400 in the five clinical isolates for mutations as compared to CDC-317. Only CPAR2_109680 (*FKS1*-ortholog) had any SNPs in our data, one synonymous and one non-synonymous (A1316T). The two crepe samples ncc-1701 and tcc-1702 both were homozygous for both SNPs and nsc-1701 was heterozygous for the non-synonymous SNP. Both were, however, absent from bsc-1700 and tsc-1702.

### Isolates Strongly Vary in Repetitive Adhesion Gene Regions

**FIGURE 4:**
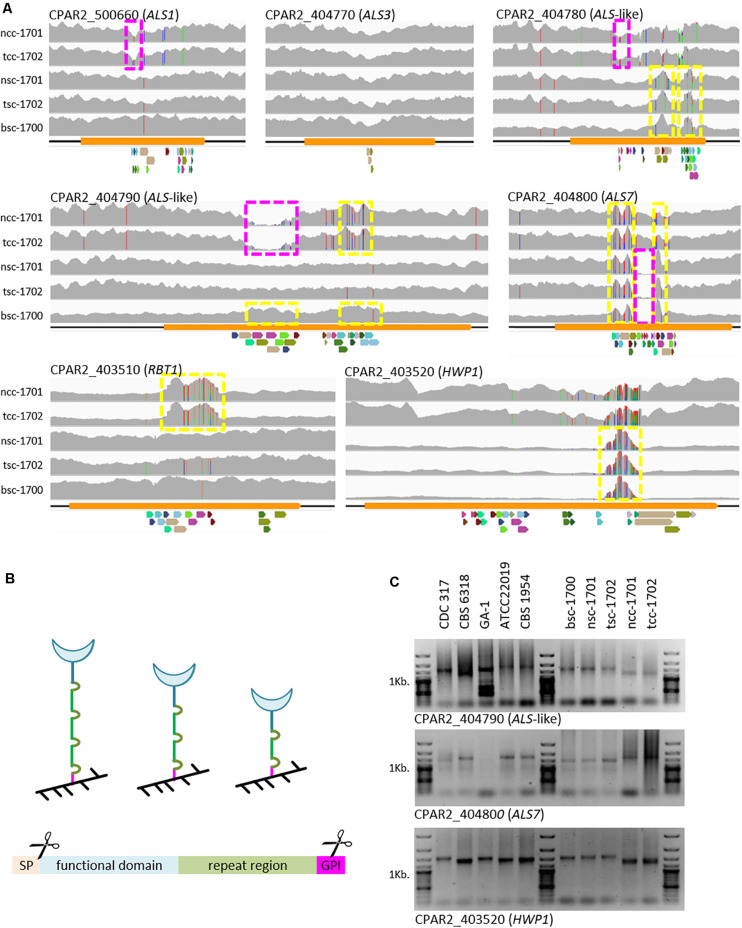
Variations in cell-wall-protein encoding genes. **(A)** Raw Illumina reads mapped onto the CDC-317 reference sequence and counted (gray, *y*-axis auto-scaled to accommodate highest read count). SNPs indicated by red, green, blue, or orange vertical dashes. Yellow boxes: increased read numbers, indicating repeat number extension; purple boxes: reduced read numbers indicating repeat number reduction. Repeats illustrated below each panel, where colors are conserved only within a panel. **(B)** GPI-cell wall proteins are covalently linked to the cell wall (black) through the C-terminal GPI domain (pink). Number variations in the repeat region (green) are thought to in- or de-crease exposure of the functional domain (blue) at the cell surface. GPI-linked cell wall proteins consist of at least four distinct domains, namely an ER-signal peptide (cleaved off), a functional domain, a repeat region, and a GPI-linkage domain (internally cleaved during anchor attachment). **(C)** Selected PCR amplicons of repetitive regions in CPAR2_404790, CAPR2_404800, and *HWP1* confirm the different lengths of the repeat regions, as suggested by whole genome sequencing.

To further explain the phenotypic properties, *C. parapsilosis* cell wall adhesin-encoding genes were analyzed to see if modification in gene-size and gene-presence varied between clinical morphotypes. In addition to a few SNPs observed, mapping of raw sequence read to the genome of CDC-317 indicated significant variation in reads numbers in the C-terminal domains of several cell wall-protein encoding genes (Figure 4A), which was also confirmed by PCR in selected cases (Figure 4C), and most strikingly for Hyphal Wall Protein encoding-gene *(HWP1)*.

## Discussion

Our initial hypothesis was that the morphologically different isolates represented different stages of the same clonal origin and started analyses in order to investigate the rate of switching; but after separation, all lineages failed to show any sign of change in morphology type across all our investigations, even despite the mixed cell shape seen microscopically in the two crepe strains. This was in contrast to the control strain ATCC 22019, whose colony appearance frequently varied and even produced a few smooth colonies in our hands.

This prompted us to investigate the genetic relationships between the five isolates by karyotyping and whole genome sequencing, and correlate these with the results of the phenotypic characterizations. Indeed both methods showed that the five strains did not have a single clonal origin but rather they belong to three different clonal groups. The degree of karyotypic changes was in accordance with those described previously, also for serial isolates (Lott et al., 1993; Shin et al., 2001; Nosek et al., 2009). The most distant group was formed by the two crepe isolates. On the genome level, the smooth isolates are more closely related to each other, but tsc-1702 is still significantly distant from the second clonal group formed by nsc-1701 and bsc-1700. This led to two possible conclusions; either the patient was colonized by at least three different clones, or these clonal groups diverged deeply within the patient. The level of sequence variation in the global *C. parapsilosis* population is relatively low as compared to other closely related species (e.g., *C. albicans*) making molecular clock calculations difficult (Pryszcz et al., 2013). Still, the two distant clonal groups of smooth vs. crepe strains were separated by >3,800 reliable SNPs, a level of divergence (Figures 3B,C) similar as that seen between fully unrelated isolates (Pryszcz et al., 2013). This makes a scenario of evolution within the patient unlikely, pointing toward co-colonization.

Because of the observed differences in biofilm formation, we specifically looked for changes in known cell wall adhesin-encoding genes. Here, we focused on genes of the *ALS* family, for which we had previously seen differences on a genome level among the reference strains used here (Pryszcz et al., 2013) and *HWP1*-like genes. Within the N-terminal functional domains only silent polymorphisms were observed (data not shown). In the repeat regions, however, we observed strong copy-number variations (Figures 4A,C). Such constellations have been documented for structural wall proteins [e.g., *C. albicans* Pir1 (Sumita et al., 2005)] as well as adhesins [*S. cerevisiae* and *C. glabrata* Epa1 (Frieman et al., 2002)], including Als-type proteins [*C. albicans* Als3 (Oh et al., 2005) and Als5 (Rauceo et al., 2006)]. The most striking amplification of repeat units observed here was found in the *HWP1* genes of the smooth strains. Although we cannot fully exclude that these differences may only reflect short-term events such as those observed in *C. albicans* (Zhang et al., 2018), together, they may at least partially explain the phenotypic differences seen between the isolates. Number variations in the repeat region of cell wall proteins are thought to in- or de-crease exposure of their functional domains on the cell surface (Figure 4B), thereby modulating their activity (Frieman et al., 2002; Oh et al., 2005; Rauceo et al., 2006). In the case of the two crepe isolates, this would indicate reduced exposure of Hwp and more strongly exposed Rbt1, a Hwp1 homolog (Figure 4A, bottom panels).

The fact that the blood culture isolate bsc-1700 was of a smooth and not of a crepe phenotype is of relevance, as biofilm formation is generally considered a virulence trait in *C. parapsilosis* (Tumbarello et al., 2012; Soldini et al., 2018). It was therefore interesting to speculate on the route of entry of this blood culture isolate. Knowing that *C. parapsilosis* is often a good biofilm former, the possibility of catheter-induced candidemia was considered likely (Escribano et al., 2014). Retrospectively, however, the culture always remained negative during the first changes of the CVCs, while yeast growth was already observed from BAL and bronchial secrete samples analyzed in close timely conjunction with the surgical intervention indicating already early colonization of the oropharynx (Table 1). However, *C. parapsilosis* was first specifically identified growing in the blood culture, and then again in the specimen taken from nose and throat in response to reporting species identification and later the susceptibility pattern. Unfortunately, the isolate obtained from the third CVC was not preserved, so that its phenotypic appearance and genetic identity must remain speculative (Table 2).

The time course of antimycotic interventions and laboratory findings (Table 1) suggests that the most likely route of entry of *C. parapsilosis* into the blood stream may be that one of the non-adhesin producing isolates was able to transgress from the respiratory tract habitat with its mixed population, which also includes bacteria, into deeper tissues and finally the sterile blood stream, either already during lung surgery or later during wound healing.

*C. albicans* Hwp1 serves as a substrate to human transglutaminase, forming a covalent bond to epithelial cells (Staab et al., 1999). Better exposure of *Cp*Hwp1 in the smooth strains, especially bsc-1700, may therefore indicate better attachment to epithelial cells *in vivo*. Dissemination may have been aided by the fact that other adhesins were not exposed sufficiently in these strains, reflected by the smooth colony morphology and low values in biofilm formation. This may have particularly placed the cells in a positions to be released from respiratory tissues into the blood stream. Since caspofungin has activity in fungal biofilms, *C. parapsilosis* may eventually have formed a biofilm on the CVC only after i.v. caspofungin therapy was stopped.

Recent data for *C. albicans* suggest that presence of multiple strains in primary non-sterile materials such as feces or epithelial swabs may be common (Zhang et al., 2018; Sitterle et al., 2019). To the best of our knowledge, we describe here for the first time co-colonization of a patient with genetically distinct *C. parapsilosis* lineages, which is in contrast to previous observations (Escribano et al., 2014) for CVC infections involving *C. parapsilosis*. Our data therefore particularly highlights the need to perform independent antimicrobial susceptibility testing on all morphotypes present on a culture plate, as they do not necessarily reflect morphologic switching of a single clonal strain but may well be the result of co-infection with multiple strains. This is also indicated by observable sequence variations within potential resistance genes, such as the 1,3-beta-D-glucan synthase subunit CPAR2_109680 (*FKS1*-ortholog). Since the non-synonymous SNP observed here is not carried by all isolates (all isolates tested here had similar MIC values for echinocandins (Table 2) within the expected test variations), and especially not in bsc-1700, which is ∼1 log 2-fold less susceptible than the others, A1316T does not appear to be a resistance-causing SNP, and it has also not been implicated by others (Garcia-Effron et al., 2008; Garcia-Effron et al., 2009).

Finally, our study also points toward a correlation of colony morphology and biofilm formation capacity and that a lack of adhesins in the cell wall could be an important step during the dissemination process. Such a model would be in strong agreement with *C. albicans*, where biofilm-dispersed yeast-form cells show significant pre-programming of virulence traits (Uppuluri et al., 2018).

## Materials and Methods

### Routine Diagnostic Procedures

Clinical specimens were plated onto Sabouraud’s GC agar and incubated at 35°C overnight. Yeast species were identified using MALDI-TOF MS (MALDI Biotyper, Bruker Daltonics) using the YOTL database (Bernhard et al., 2014). Susceptibility testing was performed on a VITEK2 system. Resistance phenotypes were confirmed using *E*-test (Biomerieux) and/or broth microdilution according to the EUCAST protocol (EUCAST, 2008).

### Strain Maintenance

Mixed cultures were differentiated on Phloxine B agar (1% yeast extract, 2% peptone, 2% glucose, 2% agar, 5 mg/ml Phloxine B) where the colonies developed stable “smooth” and “crepe” phenotypes after 96 h of incubation at 30°C (Laffey and Butler, 2005). Each stable lineage was deposited with the German Collection of Microorganisms and Cell Cultures DSMZ (Table 2).

### Biofilm Formation on Polystyrene

The capacity to form biofilms on plastic materials was analyzed in polystyrene 96 well dishes (Greiner Bio-one), as described before for *C. glabrata* (Gomez-Molero et al., 2015; Carrete et al., 2018). Briefly, cells were cultured overnight in YPD medium (1 % yeast extract, 2% peptone and 2% glucose) at 30°C in an orbital shaker at 220 rpm, the optical density adjusted to McFarland 2 and suspensions diluted fourfold to a total volume of 200 μl YPD into 96 well plates, and incubated at 37°C for 24h. Medium was removed by turning the plates upside down, tapping, and washing once with 200 μl deionized water. Attached biofilms were stained for 30 min with 100 μl 0.1% (w/v) crystal violet. Excess dye was removed by aspiration and the biofilm washed once with 200 μl deionized water. Biofilms were disrupted by pipetting up-and down in 200 μl 1% SDS in 50% ethanol. Crystal violet staining intensity was measured as the OD_490 nm_. All data shown is the average of two independent experiments with four biological replicates each.

### Microscopy

For aggregation analyses, cells were grown overnight at 30°C in 3 ml YPD medium (1% yeast extract, 2% peptone, 2% glucose) in an orbital shaker at 220 rpm, harvested, washed twice with PBS, and adjusted to an OD_600_ = 2. Aggregation was observed at 10X magnification.

For a precise differentiation of cell morphologies, the cells were harvested from overnight cultures, calcofluor-white stained, and fixed with 100% methanol. After washing twice, PBS buffered cells were embedded in Mowiol 4-88 and observed at 100X magnification.

### Agar Invasion

Development of morphotypes was scored on day 10 on Phloxine B YPD agar plates incubated at 30°C, when cells were washed off under running water. Agar invasion was classified as “no” (agar surface unaltered), “moderate” (faint imprint), or “strong” invasion (clear imprint).

### Karyotyping

Chromosomal patterns were visualized by pulsed-field gel electrophoresis as previously described for *C. glabrata* (Bader et al., 2012; Carrete et al., 2018), with adaptation only of pulse times to initial pulse time 70 s, final pulse time 140 s, and run time for 20h in 1xTAE at 200V and 17°C. Marker chromosome sizes (Mbp) of isolate CDC-317 were calculated from the respective genome sequence (version s01-m03-r25) obtained from the *Candida* Genome Database (Skrzypek et al., 2017).

### Genome Characterization

Genomic sequencing was performed at the ultra-sequencing core facility of the Center for Genome Regulation CRG, Barcelona, using Illumina 2x100, HiSeq2000 sequencing machines on paired-end libraries fragmented by nebulization in Covaris to a size of ∼600 bp. Base calling was performed using Illumina pipeline software. Raw sequencing data has been deposited in SRA (BioProject accession PRJNA516045, Table 2). Sequenced reads were mapped with the bwa mem command from BWA (Li, 2013) to CDC-317. The mapped reads were sorted and duplicates marked with picard tools (The Broad Institute, 2017). Variants were called using Freebayes (Garrison and Marth, 2012) to jointly genotype all the strains involved. Single nucleotide polymorphisms (SNPs) for which the mean mapping quality was below 30, the QUAL value was below 20, or the read depth was below 30 were removed using the vcffilter tool from vcflib (Garrison, 2020). A distance-based tree from the polymorphic SNPs was computed using BioNJ as implemented in PhyML v3.1. (Guindon et al., 2010) with default parameters. All branches received a support >88% based on 100 replicates. For Multiple Correspondence Analysis of mutations a table of allele pairs at each position in the genome that had a mutation in at least one of the included strains was created, and then an object that could be plotted using the fviz_mca_ind function in factoextra (Kassambara and Mundt, 2017) was created using the dudi.acm function in ade4 (Dray and Dufour, 2007).

Short nucleotide repeats in the CDC-317 sequence of the genes of interest were predicted using repeat finder as implemented in Geneious (Kearse et al., 2012). Changes in repeat length were confirmed by PCR, with conditions given in Table 3.

**TABLE 3:** oligonucleotides and conditions used for PCR amplification.

Name	Sequence	PCR conditions
CPAR2-404800-*ALS7*-F	5′-CCAACCACCACAGT CACAACATCT-3′	Annealing@68°C
CPAR2-404800-*ALS7*-R	5′-CTGTTGAGCCTG TAGGTGCA-3′	30 cycles 5 min
CPAR2_403520-*HWP1*-F1	5′-CTTGCTCGAAT GGTGGATGC-3′	Annealing@58°C
CPAR2_403520-*HWP1*-R1	5′-ACCGTTGTTGT CTTGATCGA-3′	30 cycles 5 min
CPAR2_404790-F1	5′-CACCACCGCAT TTTGGACTG-3′	Annealing@58°C
CPAR2_404790-R1	5′-CACCTTCCCCA GTCCAGAAC-3′	30 cycles 5 min

## Data Availability Statement

The datasets generated for this study can be found in the SRA, BioProject accession PRJNA516045.

## Ethics Statement

The manuscript was assessed by the local ethics committee of the University Medicine Göttingen and there were no ethical or legal concerns. The case report does not violate the anonymity of the patient.

## Author Contributions

EG-M, JW, and LC designed and performed the experiments. MW and AD organized routine diagnostics and aggregated patient data. EG-M, AG, UG, TG, and OB evaluated and interpreted the laboratory data. EG-M, TG, and OB wrote the manuscript. EG-M and OB conceived the study. All authors contributed to the article and approved the submitted version.

## Conflict of Interest

The authors declare that the research was conducted in the absence of any commercial or financial relationships that could be construed as a potential conflict of interest.
